# Affine transformations accelerate the training of physics-informed neural networks of a one-dimensional consolidation problem

**DOI:** 10.1038/s41598-023-42141-x

**Published:** 2023-09-20

**Authors:** Luis Mandl, André Mielke, Seyed Morteza Seyedpour, Tim Ricken

**Affiliations:** 1 Institute of Structural Mechanics and Dynamics in Aerospace Engineering, Faculty of Aerospace Engineering and Geodesy, University of Stuttgart, Pfaffenwaldring 27, 70569 Stuttgart, Germany; 2 Biomechanics Lab, Institute of Structural Mechanics and Dynamics in Aerospace Engineering, Faculty of Aerospace Engineering and Geodesy, University of Stuttgart, Pfaffenwaldring 27, 70569 Stuttgart, Germany

**Keywords:** Computational science, Scientific data, Civil engineering, Mechanical engineering

## Abstract

Physics-informed neural networks (PINNs) leverage data and knowledge about a problem. They provide a nonnumerical pathway to solving partial differential equations by expressing the field solution as an artificial neural network. This approach has been applied successfully to various types of differential equations. A major area of research on PINNs is the application to coupled partial differential equations in particular, and a general breakthrough is still lacking. In coupled equations, the optimization operates in a critical conflict between boundary conditions and the underlying equations, which often requires either many iterations or complex schemes to avoid trivial solutions and to achieve convergence. We provide empirical evidence for the mitigation of bad initial conditioning in PINNs for solving one-dimensional consolidation problems of porous media through the introduction of affine transformations after the classical output layer of artificial neural network architectures, effectively accelerating the training process. These affine physics-informed neural networks (AfPINNs) then produce nontrivial and accurate field solutions even in parameter spaces with diverging orders of magnitude. On average, AfPINNs show the ability to improve the ${\mathscr {L}}_2$ relative error by $64.84\%$ after 25,000 epochs for a one-dimensional consolidation problem based on Biot’s theory, and an average improvement by $58.80\%$ with a transfer approach to the theory of porous media.

## Introduction

The accurate prediction of the time-dependent behavior of poroelastic porous media is a fundamental challenge in geotechnical and geological engineering, its main purpose being the optimization of designs and engineering safety assurance^1^. In the context of porous media, consolidation is the dissipation of pore fluid pressure through the solid matrix induced by changes in stress^2^. Theories of consolidation can be classified into two groups, namely coupled and uncoupled theories. In uncoupled theories, the total stress remains constant everywhere throughout the consolidation process, and strains are affected only by changes in pore pressure^3,4^. In coupled theories, the interaction between solid matrix and pore fluid is included in the formulation, leading to more complex partial differential equations (PDEs) governing displacements and pore fluid pressure^5,6^. Most coupled theories use homogenization approaches. Common models using homogenization are Biot’s theory^5^, the theory of mixtures^7,8^, and the theory of porous media (TPM)^9–11^. The models differ primarily in the point of introduction of homogenized quantities^12^. The TPM provides a robust and thermodynamically consistent^13–15^ framework to describe a macroscopic continuum-mechanical model of multiphase, multicomponent fluid-saturated porous media. Soil mechanics^16–19^, environmental engineering^20–23^, and continuum biomechanics^24–27^ are examples of engineering disciplines where the TPM was successfully employed to solve problems via single-scale macroscopic homogenization approaches. Machine-learned surrogate models can be employed to produce efficient solution approximations within this framework even for complex simulations^28^. The primary issue with coupled models is their instability. Their direct numerical integration using the standard finite element method (FEM) often results in suboptimal solutions^29–31^. Even for the simplest soil consolidation problem, numerical models based on coupled formulations are ill-conditioned when the soil permeability is low, or when the soil deformability is large^32^. Hence, even in uncoupled theories, analytical approaches are still widely utilized to deal with soil consolidation problems in general^2^. Various robust analytical approaches have been developed for different consolidation problems, including large strain consolidation^33^, unsaturated soils with vertical loading^34^, consolidation problems in multi-layered soil^35^, consolidation equations with different boundary conditions^36^, as well as the consolidation of a column with permeable top and impermeable bottom (PTIB) under general transient loading^6^.

More recently, physics-informed or knowledge-guided machine learning has emerged as a highly promising method in the scientific community for merging data-driven methods with domain knowledge^37,38^. By including the governing equations of a problem (or other types of information, such as symmetry), the solution space is restricted to fields that satisfy these conditions. Consequently, the amount of data required for training can be reduced, since the imposed properties no longer need to be deducted from the data. Instead, they are incorporated as prior knowledge^37,39^. Among the wide range of different machine learning methods^39^, physics-informed neural networks (PINNs)^40^ are particularly suitable for addressing typical engineering problems, since the structure of problem-describing differential equations and the corresponding specific boundary conditions is analogous to the structure in numerical or analytical solution techniques. It has been known since 1998^41^ that differential equations can be modeled using a combination of artificial neural networks’ (ANNs)^40–42^, their universal approximation capabilities (cf. Cybenko^43^, Hornik^44^, Maiorov and Pinku^45^, and Kidger and Lyons^46^), and automatic differentiation^47^. However, this method’s enormous potential with modern computing methods has only recently been reestablished^40^. This straightforward, yet versatile concept has been applied to various problems from all fields of engineering, e.g., incompressible Navier–Stokes equations^48^, heat transfer^49^, systems biology^50^, and subsurface flow^51^. Gradient pathologies, where the separate terms in gradient-based optimization operate against each other, and propagation failures^52,53^ are well-known challenges due to the composite construction of the loss function in PINNs^54^. In the latter case, the solution adhering to the boundary values cannot be propagated into the inner field, resulting in the discovery of trivial solutions which technically satisfy the PDE residual. These issues also arise in coupled problems, e.g., in poromechanical consolidation^55,56^, leading to convergence problems. PINNs have been utilised to develop surrogates for numerous processes within porous media. For instance, immiscible two-phase fluid transport in porous media, where the fluid flux results in shocks and rarefactions^57^. Bekele^58^ utilized PINNs to solve Barry-Mercer’s problem^59^ which models deformation due to a fluid source in the domain. The uncoupled theory of consolidation where only pore water pressure is solved has been studied both in one dimension for a permeable top and permeable bottom^60^ as well as in two dimensions by modeling consolidation with drained top boundary in one direction, and both drained top and bottom boundary in the other direction^61^. Nonlinear diffusivity and Biot’s theory were addressed by Kadeethum et al.^62^ for isotropic and homogeneous porous media, where nonlinear diffusivity is a special variant of Biot’s theory with decoupled balance of linear momentum and balance of mass, hence, only solving for fluid pressure. All physical constants were set to 1.0 for both nonlinear diffusivity and Biot’s equation, where the latter encapsulates Dirichlet and Neumann boundary conditions. Haghighat et al.^63^ demonstrated that combining a dimensionless form for fully and partially saturated flow in porous media with a sequential training strategy and adaptive weighting methods improves stability. The authors primarily demonstrate how a sequential stress-split training strategy, comparable to sequential techniques used for simulation with finite elements, improves robustness and convergence at the expense of additional computational resources in the form of longer training. Effectively, the training process is separated for each output variable by using independent ANNs, cf.^64^, where in each iteration, first the pressure and its network are optimized and evaluated before the different quantities, i.e., displacement and strain, and the respective networks are updated and evaluated. This ansatz is then used for the Mandel problem, which describes the deformation of a fluid-saturated, poroelastic rectangular domain under load from top and bottom by using Biot’s theory^65^, Barry-Mercer’s problem, and a two-phase drainage problem, where fluid drains from the bottom of a solid column due to gravity with a gas phase at the top. The proposed method performed well in all three problems with a customized PINN setup^63^. Similarly, Amini et al.^66^ used PINNs with nondimensionalized equations for thermo-hydro-mechanical processes in porous media alongside a sequential training strategy while using adaptive weighting strategies to further facilitate the difficulties in training PINNs due to the nature of the optimization problem with competing terms in the composite loss function, mentioned by several authors^53,54,62,63,66–69^. Based on the balances of linear momentum, mass, and energy alongside Darcy’s law for fluid flow and Fourier’s law for heat transfer, Amini et al.^66^ use this approach to model various setups, including a one-dimensional consolidation problem with displacement, pressure, and temperature as variables.

In this work, we show how affine transformations in the output layer of ANNs accelerate the convergence behavior of PINNs. For this purpose, we use Biot’s theory of porous media to demonstrate the behavior through a simplified theory with an analytical solution and the TPM as a more complex problem with a numerical solution. To our best knowledge, this is the first solution of a TPM problem using PINNs.

The paper is structured in the following manner: In the “Methods” section, we explain the rationale for the chosen consolidation problem itself and the potential challenges for training PINNs on this problem. In the “Results and discussion” sections, we present how we chose parameters for the affine transformations and compare vanilla PINNs to AfPINNs and discuss improvements in mean and standard deviation for loss, absolute deviations of field variables, and the ${\mathscr {L}}_2$ relative error with the remaining hyperparameters held constant. Furthermore, we discuss the number of epochs necessary to surpass given lower limits for both variants. Moreover, we conjecture reasons for the behavior discovered in this work and discuss potential future applications.

## Methods

We start by explaining the concept of PINNs before motivating the addition of affine transformations. After a subsequent formalization of including affine transformations in PINNs, we explain similarities to other well-known techniques in physics-informed machine learning. We will then describe the chosen one-dimensional consolidation problem using both Biot’s theory and the TPM, including the respective reference solutions, their characteristics, and typical challenges when dealing with this type of consolidation problem. We conclude this section with the base setting for all training processes used within this work as we combine PINNs and the PDEs underlying the consolidation problem.

### Physics-informed neural networks

PINNs are ANNs constrained by a differential equation. The fundamental idea is that the physical laws described by the differential equation are added to the loss term in addition to the classical loss function, restricting the ANN solutions to those satisfying these laws. Considering the general nonlinear partial differential equation on the bounded domain $\Omega \subseteq \mathbb R^n$, the common notation is given as

1 $$\begin{aligned} \frac{\partial {\textbf{u}}({\textbf{x}},t)}{\partial t}+{\mathscr {N}}[{\textbf{u}}({\textbf{x}},t);\pmb {\lambda }]={\textbf{0}},~{\textbf{x}} \in \Omega ,~t \in [0,T], \end{aligned}$$

with the field solution ${\textbf{u}}({\textbf{x}},t)$ and the parameterized nonlinear differential operator ${\mathscr {N}}[\cdot ]$ with parameters $\pmb {\lambda }$. Since we can treat ANNs as universal approximators, we may use one to approximate the solution ${\textbf{u}}({\textbf{x}},t)$. The notation can be compactified further by integrating the temporal component into ${\textbf{x}}$, yielding an arbitrary spatiotemporal nonlinear differential operator ${\mathscr {N}}^*[\cdot ]$ on the spatiotemporal domain $\Omega ^*\subseteq {\mathbb {R}}^{n+1}$. Additionally, we can introduce BCs on the boundary of our domain $\partial \Omega ^*$ with a general differential operator ${\mathscr {B}}[\cdot ]$ for representing different types of BCs, e.g., Dirichlet and Neumann BCs given as ${\textbf{b}}({\textbf{x}})$:

2 $$\begin{aligned} {\mathscr {N}}^*[{\textbf{u}}({\textbf{x}});\pmb {\lambda }]&={\textbf{0}},~{\textbf{x}} \in \Omega ^*, \end{aligned}$$

3 $$\begin{aligned} {\mathscr {B}}[{\textbf{u}}({\textbf{x}});\pmb {\lambda }]&={\textbf{b}}({\textbf{x}}),~{\textbf{x}} \in \partial \Omega ^*. \end{aligned}$$

We can utilize automatic differentiation for the derivatives. Since all operations (addition, multiplication, and nonlinear activation function) and their derivatives are known, they can be inferred from the computational graph. This allows the general network training with backpropagation and additionally allows to get the derivative of the output of the ANN with respect to the inputs to an arbitrary order. For the sake of brevity in the mathematical descriptions, we abbreviate the PDE as

4 $$\begin{aligned} {\textbf{f}}({\textbf{x}}){:}{=}{\mathscr {N}}^*[{\textbf{u}}({\textbf{x}});\pmb {\lambda }]. \end{aligned}$$

An ANN in a PINN setting simultaneously approximates the field solution and its derivatives, thereby reconstructing the PDE itself, which is used as an additional regularization term in the loss during training (see Eqs. 5 and 7). Through the addition of regularization terms (as shown in Eqs. 5–8), the ANN can be influenced by data in the form of boundary conditions, field variables, and additionally by physical laws in the form of differential equations. The former directly allows for the integration of Dirichlet as well as higher-order BCs into the scheme through the differential operator ${\mathscr {B}}[\cdot ]$. Without loss of generality, we adapt this general notation for the construction of the loss function by directly introducing Dirichlet and Neumann BCs in the loss function for two reasons: First, to keep the theoretical formulation closer to the implementation and secondly, to prepare for later use in one-dimensional consolidation. In this work, we take the mean square error (MSE) loss for Dirichlet BCs on ${\textbf{u}}({\textbf{x}})$, the PDE itself through (${\textbf{f}}({\textbf{x}})$), and Neumann BCs on $\nabla _{{\textbf{n}}}{\textbf{u}}({\textbf{x}})$. By introducing the network predictions as a function $\hat{{\textbf{u}}}({\textbf{x}}_i;\pmb {\theta })$ parameterized by the input ${\textbf{x}}$ and the collection of shared weights and biases $\pmb {\theta }$, we obtain the following loss:

5 $$\begin{aligned} {\mathscr {L}}&= {w_u}{\mathscr {L}}_u + {w_f}{\mathscr {L}}_f + {w_{u_n}}{\mathscr {L}}_{u_n}, \end{aligned}$$

6 $$\begin{aligned} {\mathscr {L}}_u&=\frac{1}{N_u}\sum _{i=1}^{N_u}\left( \hat{{\textbf{u}}}({\textbf{x}}_i^{(u)};\pmb {\theta })-{\textbf{u}}_i\right) ^2, \end{aligned}$$

7 $$\begin{aligned} {\mathscr {L}}_f&=\frac{1}{N_f}\sum _{i=1}^{N_f}\left( \hat{{\textbf{f}}}({\textbf{x}}_i^{(f)};\pmb {\theta })-{\textbf{f}}_i\right) ^2=\frac{1}{N_f}\sum _{i=1}^{N_f}\left( \hat{{\textbf{f}}}({\textbf{x}}_i^{(f)};\pmb {\theta })\right) ^2, \end{aligned}$$

8 $$\begin{aligned} {\mathscr {L}}_{u_n}&=\frac{1}{N_{u_n}}\sum _{i=1}^{N_{u_n}}\left( \nabla _{\textbf{n}}\hat{{\textbf{u}}}({\textbf{x}}_i^{({u_n})};\pmb {\theta })-\nabla _{\textbf{n}}{\textbf{u}}_{n,i}\right) ^2, \end{aligned}$$

where for each term different collocation points are sampled, indicated by the superscripts as being sampled for Dirichlet BCs (${\textbf{x}}_i^{(u)}$), the PDE evaluation itself (${\textbf{x}}_i^{(f)}$), and Neuman BCs (${\textbf{x}}_i^{u_n}$). The hat (${\hat{\cdot }}$) indicates quantities approximated by the ANN. Furthermore, $w_u$, $w_f$, and $w_{u_n}$ are weights that can be adapted to change the influence of the respective terms in the cumulative loss function^67,68^. Within this work, we set $w_u = w_f = w_{u_n} = 1$, i.e., we do not utilize loss weighting techniques. While other types of ANN architectures such as convolutional or recurrent neural networks have been combined with physics-informed machine learning, see for example the overview by Cuomo et al.^67^, a widespread comparative study of the approximation capabilities is to the best knowledge of the authors not present. Hence, within this work, only fully-connected neural networks are used.

### Affine transformations for physics-informed neural networks

In principle, given the requirements for a universal approximator, every ANN has the potential to adapt to some target function within the available function space. Due to the training process, which is gradient-based and attributes approximation errors to weights from the last to the first layer, this process can be slowed down. This happens, for example, by the internal covariate shift^70^ or generally small gradients in the first layers. Our basic idea for using affine transformations was to give the network a bias for the location and width of the initial distribution, which is as close as possible to the desired distribution. Since these distributions may be in different orders of magnitude and locations for each output quantity, an offset factor *b* and a scaling factor *w* are specified for each quantity, summarized as vectors ${\textbf{w}}$ and ${\textbf{b}}$. This can be understood as an affine transformation layer. ANNs with affine transformations (AfNNs) in the output can straightforwardly be used in PINNs. The only component added by using the affine layer is a scaling of the gradients with the factors ${\textbf{w}}$ in the last layer of the underlying ANN. All fundamental operators are known, so arbitrary derivatives of outputs with respect to the inputs can be computed. We call this an affine physics-informed neural network (AfPINN). The affine transformations thus influence the general setup in two ways: Scaling and offset factors cause a change in the initial distributions of the output variables, and the factors additionally scale the gradients during the training with backpropagation. Hence, in the optimal setup, AfNNs and AfPINNs are not only initially closer to the desired solution, but also optimized gradients accelerate the training process. For a multivariate case, this implies a coupled behavior of the hyperparameters of the optimization method employed, such as the learning rate, and the transformation parameters. In addition, in the multidimensional case, there is the possibility of distributing gradients along the different optimization directions. This can help in optimization with competing components in the aggregated loss function since distorted loss landscapes are made more regular by scaling (cf. Krishnapriyan et al.^69^). Conversely, learning rate and scaling factors are directly correlated in the univariate case, so that they influence each other. Hence, we keep the learning rate fixed throughout this work. For the sake of clarity, we want to emphasize that scaling and offset factors of the affine layer are hyperparameters, whereas the weights and biases of the other layers within the ANN are optimized using a stochastic optimization algorithm, e.g., a variant of gradient descent. The differences between the affine layer and the classical fully-connected layer are the lack of a nonlinear activation function and the fact that each neuron has only one connection to the output of the previous layer, i.e., one associated input neuron. A similarity of the idea to batch normalization and its aim to reduce the internal covariate shift as laid out by Ioffe and Szegedy^70^ is recognizable. Batch normalization aims to normalize the output values after each layer by recentering and rescaling. This prevents changes in previous layers induced by changes in the input distribution in subsequent layers, thus accelerating training. However, batch normalization tries to enforce output distributions with a mean of 0 and a standard deviation of 1. In order to predict values in a domain with previously unspecified bounds, which can occur in the solution of PDEs, an unrestricted prediction must be possible at least in the output layer. Here, affine transformations in the output layer are seen as complementary to batch normalization.

### One-dimensional consolidation with sine loading using Biot’s theory

**Figure 1:**
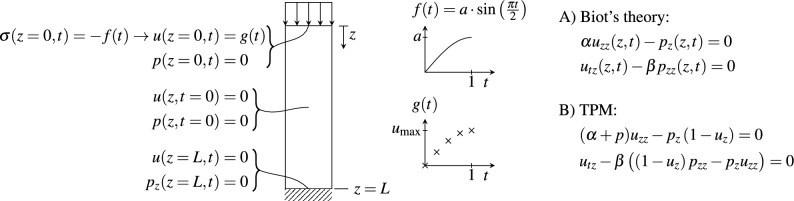
Sketch of the PTIB column with the initial and boundary conditions including the load profile for generating an analytical solution and the variation used for physics-informed neural networks. The latter uses Dirichlet BC for displacement on the top, whereas the first uses a Dirichlet BC for stress. The displacement BC for PINNs can be sampled from the analytical solution *g*(*t*) for the displacement at $z=0$. The analytical solution is calculated based on the prescribed stress $\sigma (z=0,t)$ as explained in the section on the analytical solution for Biot’s theory. Furthermore, Biot’s theory (A) and TPM (B), which both describe behaviour of the PTIB column within this paper are given in short notation on the right.

The one-dimensional problem, as for example derived by Stickle and Pastor^6^, consists of an initially unloaded PTIB column of length *L* with a load applied on the perfectly drained upper boundary. Every movement is one-dimensional, i.e., displacement occurs in the *z*-direction, normal to both the upper and lower boundary, where the latter is fixed and impermeable (cf. Fig. 1). The governing equations are given as:

9 $$\begin{aligned} (\lambda +2\mu )\frac{\partial ^2 u(z,t)}{\partial z^2}-\frac{\partial p(z,t)}{\partial z}&=0, \end{aligned}$$

10 $$\begin{aligned} \frac{\partial ^2 u(z,t)}{\partial t\partial z} - \frac{k}{\rho g}\frac{\partial ^2 p(z,t)}{\partial z^2}&=0, \end{aligned}$$

where *u* is the (vertical) displacement, *p* is the pore fluid pressure, *z* is the vertical position, *t* is the time, $\lambda$ and $\mu$ are the Lamé constants, *k* is Darcy’s permeability, $\rho$ is the fluid density, and *g* is the gravitational acceleration. Since we do not focus on the meaning of the parameters in the context of this work but only consider their orders of magnitude and influence on the results, they are replaced by two factors $\alpha$ and $\beta$:

11 $$\begin{aligned} \alpha \frac{\partial ^2 u(z,t)}{\partial z^2}-\frac{\partial p(z,t)}{\partial z}&=0, \end{aligned}$$

12 $$\begin{aligned} \frac{\partial ^2 u(z,t)}{\partial t\partial z} - \beta \frac{\partial ^2 p(z,t)}{\partial z^2}&=0. \end{aligned}$$

The respective boundary and initial conditions for this problem are:

13 $$\begin{aligned} u(z, t=0)&=0, \end{aligned}$$

14 $$\begin{aligned} p(z, t=0)&=f(0)=0, \end{aligned}$$

15 $$\begin{aligned} \sigma (z=0, t)=-f(t)=&-a\cdot \sin \left( \frac{\pi t}{2}\right) , \end{aligned}$$

16 $$\begin{aligned} p(z=0,t)&=0, \end{aligned}$$

17 $$\begin{aligned} u(z=L, t)&=0, \end{aligned}$$

18 $$\begin{aligned} p_z(z=L, t)&=0, \end{aligned}$$

where Eq. (15) defines the loading pattern. This loading pattern consists of a stress boundary to obtain the analytical solution from which we can sample pressure and displacement values for the adapted problem which will be used in PINNs and AfPINNs. In the following, we will only consider the time frame $t\in [0~\text {s},~t_{\text {end}}=1~\text {s}]$ and the length $z\in [0~\text {m},~L=1~\text {m}]$. Hence, the loading pattern consists of the first quarter of a sine period. Figure 1 shows the consolidation problem with its boundary and initial conditions for the analytical solution and a variant that will be used for PINNs.

#### Analytical solution

Following the work of Stickle and Pastor^6^, we can derive an analytical solution for the given problem setup and load for Biot’s theory. The authors’ work presents a scheme based on the evaluation and subsequential summation of series elements for pressure to obtain the global evolution of pore pressure and vertical displacement.

19 $$\begin{aligned} p(z, t)&= \sum _{n=0}^{\infty }p_n(t)\sin \left( \frac{(1+2n)\pi z}{2L}\right) , \end{aligned}$$

20 $$\begin{aligned} u(z, t)&= \frac{f(t)(L-z)}{\alpha }-\frac{1}{\alpha }\sum _{n=0}^{\infty }\frac{2L}{(1+2n)\pi }p_{n}(t)\cos \left( \frac{(1+2n)\pi z}{2L}\right) . \end{aligned}$$

The individual series elements result from the solution of the related Sturm-Liouville eigenvalue problem of the homogeneous problem, since the pore pressure can be expressed as a linear combination of the eigenfunctions. Substituting the solution of the homogeneous problem into the original problem alongside the use of $f(0)=0$ for the load function $f(t)=a\cdot \sin \left( \frac{\pi t}{2}\right)$ yields the following expression for the series elements:

21 $$\begin{aligned} p_{n}(t) = \frac{4}{\pi (2n+1)}\left[ e^{-{\mathfrak {N}}_nt}f(0)+\int _0^t e^{-{\mathfrak {N}}_n(t-\tau )}f'(\tau )\text {d}\tau \right] =\frac{4}{\pi (2n+1)}\int _0^t e^{-{\mathfrak {N}}_n(t-\tau )}f'(\tau )\text {d}\tau . \end{aligned}$$

The integral included in the solution of the inital value problem for the selected load function is given by

22 $$\begin{aligned} \int _0^t e^{-{\mathfrak {N}}_n(t-\tau )}f'(\tau )\text {d}\tau =\frac{\pi a \left( -2{\mathfrak {N}}_ne^{-{\mathfrak {N}}_nt}+2{\mathfrak {N}}_n\cos \left( \frac{\pi t}{2}\right) +\pi \sin \left( \frac{\pi t}{2}\right) \right) }{4{\mathfrak {N}}_n^2+\pi ^2}, \end{aligned}$$

with ${\mathfrak {N}}_n=\alpha \beta \left( \frac{(1+2n)\pi }{2L}\right) ^2$ and the series index *n*. We found using 10,000 series elements to suffice in precision for our purposes. This type of displacement-driven consolidation may yield pressure spikes at the start of loading, as typically $\beta \ll \alpha$, so the first load is almost completely converted into pore fluid pressure as the solid skeleton has a higher resistance. This phenomenon is mitigated in part through low load increments by the sine function. Furthermore, significantly different orders of magnitude can appear in coupled pressure-displacement problems. High values of $\alpha$ are associated with small displacements and low values of $\beta$ are associated with large pressures.

### One-dimensional consolidation with sine loading using the theory of porous media

We utilize a one-dimensional form of the TPM, which can be derived from the full set of equations of a two-phasic, incompressible, and isothermal continuum while neglecting volume forces, for example described by Bertrand et al.^71^. These equations are given as:

23 $$\begin{aligned} (\lambda +2\mu )\frac{\partial ^2 u(z,t)}{\partial z^2}-\frac{\partial p(z,t)}{\partial z}\left( 1-\frac{\partial u(z,t)}{\partial z}\right) +p(z,t)\frac{\partial ^2 u(z,t)}{\partial z^2}&=0, \end{aligned}$$

24 $$\begin{aligned} \frac{\partial ^2 u(z,t)}{\partial t\partial z} - k\left( \left( 1-\frac{\partial u(z,t)}{\partial z}\right) \frac{\partial ^2 p(z,t)}{\partial z^2}-\frac{\partial p(z,t)}{\partial z}\frac{\partial ^2 u(z,t)}{\partial z^2}\right)&=0, \end{aligned}$$

with (vertical) displacement *u*, pore fluid pressure *p*, vertical position *z*, time *t*, Lamé constants $\lambda$ and $\mu$, and Darcy permeability *k*. Once again introducing factors $\alpha$ and $\beta$ while reordering terms, we obtain the equations

25 $$\begin{aligned} (\alpha +p)\frac{\partial ^2 u(z,t)}{\partial z^2}-\frac{\partial p(z,t)}{\partial z}\left( 1-\frac{\partial u(z,t)}{\partial z}\right)&=0, \end{aligned}$$

26 $$\begin{aligned} \frac{\partial ^2 u(z,t)}{\partial t\partial z} - \beta \left( \left( 1-\frac{\partial u(z,t)}{\partial z}\right) \frac{\partial ^2 p(z,t)}{\partial z^2}-\frac{\partial p(z,t)}{\partial z}\frac{\partial ^2 u(z,t)}{\partial z^2}\right)&=0. \end{aligned}$$

The same boundary conditions as for Biot’s theory are used (see Eqs. 13–18). Similarly, we use data points from the reference solution for the displacement at the upper boundary to obtain a displacement-driven problem, as outlined in Fig. 1. The reference solution was obtained by simulating of a two-dimensional column with the finite element method as described by Bertrand et al.^71^. The simulation was done using FEniCS^72^ with the DOLFINx solver^73^ with 1000 elements in and 100 elements perpendicular to the consolidation direction for ${\textbf{x}} = (z,x)$ with $z\in [0~\text {m},~1~\text {m}]$ and $x\in [0~\text {m},~0.1~\text {m}]$, and 1000 time steps for $t \in [0~\text {s},~1~\text {s}]$. The traction boundary was given with a maximum of $a=0.1$, while the equation constants were fixed to $\alpha =\beta =1$ as was done for Biot’s theory. Taylor-Hood elements were used for discretization. Only the central row at the symmetry axis was extracted to obtain the one-dimensional reference solution. Figure S.1 in the supplementary material compares displacement and pore fluid pressure for Biot’s theory and the TPM with the given settings, i.e., identical loading, boundary conditions, and equation parameters. Using the TPM results in a larger absolute displacement, while the pressure decays somewhat more gradually compared to using Biot’s theory with identical parameters. The global behavior is similar, but the TPM shows a different behavior towards the end of the simulation time due to additional nonlinear terms.

### AfPINNs for consolidation problems

**Figure 2:**
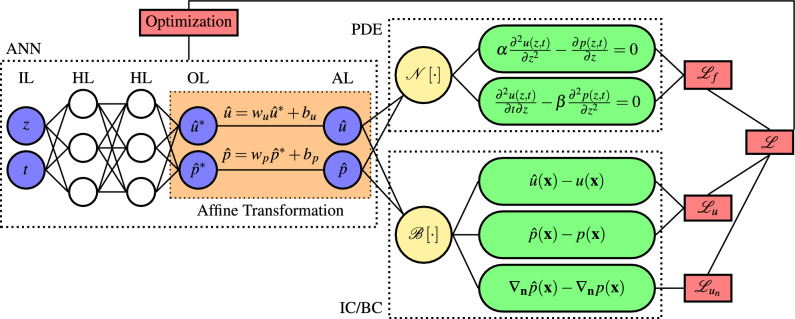
Affine physics-informed neural network set up for one-dimensional consolidation using Biot’s theory including boundary and initial conditions with Neumann- and Dirichlet-type conditions subsumed under the boundary condition operator ${\mathscr {B}}[\cdot ]$. The losses alongside the optimization loop are depicted with red boxes. The ANN is depicted with input layer (IL), hidden layers with nonlinear activation (HL), output layer with linear activation (OL), and affine layer (AL) with the affine transformations encapsulated in orange.

Figure 2 shows the setup of AfPINNs used for the one-dimensional consolidation problem using Biot’s theory. In an analogous approach for the TPM, the equation terms within the PDE box in the upper right are adjusted for the TPM. The remaining parts are unchanged.

### Training AfPINNs for one-dimensional consolidation

The base hyperparameters for all AfPINNs were 12 hidden layers with 40 neurons and *tanh* activation, 2 input neurons (for *z* and *t*) , 2 output neurons (for displacement *u* and pressure *p*) with linear activation, 100 sample points on the BCs, 100 sample points on the ICs, and 1000 collocation points within the domain. Training was performed for 25,000 epochs using ADAM optimization^74^ with a fixed learning rate of $10^{-3}$ using DeepXDE^75^ on the full data batch. All weights and biases were initialized using the Glorot normal initializer^76^.The underlying problem strongly encourages trivial solutions, especially for the pressure field, because no non-zero values are introduced for the pressure. Both initial and boundary conditions are given as Dirichlet- or Neumann-type conditions with fixed values of 0. In contrast, the displacement field, whose values differ from 0, is given as a Dirichlet boundary condition. In addition, even the order of magnitude is given, because in the shown problem, the largest displacement is at the upper edge, i.e., exactly where the displacement is prescribed. Accordingly, the network must learn that non-zero values for the pressure field may occur and the numerical size of the maximum value must be identified, which may lie outside of the predefined initialization range. Therefore, the particular values of the pressure field can be derived from the PDE system alone. This is an obstacle per se for the underlying problem since even trivial solutions, i.e., $u(z,t) = p(z,t) = 0$ for arbitrary *z* and *t*, satisfy all initial and boundary conditions and the PDEs, except for the displacement boundary condition for *u*(0, *t*). Hence, an additional obstacle arises in the optimization problem since not only must correct values be found that satisfy all constraints, including the PDEs, but also trivial solutions must be averted. Thus, a major problem is generating and propagating the correct solution into the field. Due to the composition of the loss function, terms with a larger value are strongly favored during training. The further the values have to leave the initialization range to represent the true solution, the geometrically closer the optimization is in the direction of the trivial solution since here, only the unoptimized partial term of the displacement boundary condition remains non-zero at the open end. Based on the linearized strain used, we move in a small-strain region, so that $\frac{u_{\text {max}}}{L}\ll 1$ holds. Gradient-based optimization entails the risk that local minima cause significant problems. In the case of PINNs, it may be necessary to tolerate large approximated field values after some initialized order of magnitude before converging to the correct solution, which comes from erroneous values from boundary conditions or field values during the optimization. This region of high interim values in the optimization between initial and sought-for solution obstructs the path of the optimization in the loss landscape. Accordingly, gradient-based optimization likely fails and yields a trivial solution. This must be addressed using either an adapted gradient-based method or an adjusted initialization scheme to either choose a feasible starting point or to change the optimization landscape. The method provided here aims for the latter two by implementing affine transformations at the ANN’s output layer.

## Results and discussion

Our main findings can be separated into three parts. First, an initial study of the influence of scaling and offset values in AfPINNs for the one-dimensional consolidation problem using Biot’s theory, as described in the “Methods” section, is presented. Secondly, from the initial study, a subsequent comparison of vanilla PINNs and AfPINNs for Biot’s Theory with optimal scaling and offset values from the initial study are compared. Lastly, a transfer learning approach using the chosen affine transformation parameters without considering other prior knowledge of the AfPINNs for the consolidation problem using the TPM is done, showing the flexibility of our method.

### Influence of affine transformations on a one-dimensional consolidation problem with Biot’s theory

**Table 1:** The 15 best combinations of scaling ($w_u$ and $w_p$) and offset parameters ($b_u$ and $b_p$) for $\alpha =\beta =1$, based on the ${\mathscr {L}}_2$ relative error.

$w_u$	$w_p$	$b_u$	$b_p$	${\mathscr {L}}_2$ rel. err.	max(abs($u-\hat{u}$))	max(abs($p-\hat{p}$))
$1\textrm{e}{-}2$	$1\textrm{e}{-}2$	$1\textrm{e}{-}5$	$1\textrm{e}{-}4$	$1.00\textrm{e}{-}2$	$2.54\textrm{e}{-}4$	$1.34\textrm{e}{-}3$
$1\textrm{e}{-}2$	$1\textrm{e}{-}2$	$1\textrm{e}{-}3$	$1\textrm{e}{-}1$	$1.11\textrm{e}{-}2$	$2.27\textrm{e}{-}4$	$1.77\textrm{e}{-}3$
$1\textrm{e}{-}3$	$1\textrm{e}{-}2$	$1\textrm{e}{-}5$	$1\textrm{e}{-}5$	$1.14\textrm{e}{-}2$	$2.64\textrm{e}{-}4$	$1.76\textrm{e}{-}3$
$1\textrm{e}{-}2$	$1\textrm{e}{-}3$	$1\textrm{e}{-}5$	$1\textrm{e}{-}1$	$1.15\textrm{e}{-}2$	$2.73\textrm{e}{-}4$	$1.37\textrm{e}{-}3$
$1\textrm{e}{-}2$	$1\textrm{e}{-}2$	$1\textrm{e}{-}4$	$1\textrm{e}{-}5$	$1.15\textrm{e}{-}2$	$4.41\textrm{e}{-}4$	$1.57\textrm{e}{-}3$
$1\textrm{e}{-}2$	$1\textrm{e}{-}2$	$1\textrm{e}{-}4$	$1\textrm{e}{-}2$	$1.18\textrm{e}{-}2$	$2.93\textrm{e}{-}4$	$1.50\textrm{e}{-}3$
$1\textrm{e}{-}2$	$1\textrm{e}{-}2$	$1\textrm{e}{-}2$	$1\textrm{e}{-}3$	$1.20\textrm{e}{-}2$	$5.12\textrm{e}{-}4$	$1.55\textrm{e}{-}3$
$1\textrm{e}{-}2$	$1\textrm{e}{-}2$	$1\textrm{e}{-}3$	$1\textrm{e}{-}2$	$1.22\textrm{e}{-}2$	$5.57\textrm{e}{-}4$	$1.47\textrm{e}{-}3$
$1\textrm{e}{-}2$	$1\textrm{e}{-}2$	$1\textrm{e}{-}5$	$1\textrm{e}{-}5$	$1.22\textrm{e}{-}2$	$4.52\textrm{e}{-}4$	$1.71\textrm{e}{-}3$
$1\textrm{e}{-}2$	$1\textrm{e}{-}2$	$1\textrm{e}{-}2$	$1\textrm{e}{-}1$	$1.22\textrm{e}{-}2$	$3.57\textrm{e}{-}4$	$1.29\textrm{e}{-}3$
$1\textrm{e}{-}2$	$1\textrm{e}{-}2$	$1\textrm{e}{-}3$	$1\textrm{e}{-}3$	$1.24\textrm{e}{-}2$	$2.64\textrm{e}{-}4$	$1.60\textrm{e}{-}3$
$1\textrm{e}{-}2$	$1\textrm{e}{-}2$	$1\textrm{e}{-}4$	$1\textrm{e}{-}4$	$1.24\textrm{e}{-}2$	$2.92\textrm{e}{-}4$	$1.83\textrm{e}{-}3$
$1\textrm{e}{-}2$	$1\textrm{e}{-}2$	$1\textrm{e}{-}2$	$1\textrm{e}{-}2$	$1.25\textrm{e}{-}2$	$3.27\textrm{e}{-}4$	$1.64\textrm{e}{-}3$
$1\textrm{e}{-}2$	$1\textrm{e}{-}3$	$1\textrm{e}{-}3$	$1\textrm{e}{-}1$	$1.26\textrm{e}{-}2$	$7.50\textrm{e}{-}4$	$1.39\textrm{e}{-}3$
$1\textrm{e}{-}2$	$1\textrm{e}{-}3$	$1\textrm{e}{-}4$	$1\textrm{e}{-}1$	$1.27\textrm{e}{-}2$	$4.92\textrm{e}{-}4$	$1.55\textrm{e}{-}3$

Maximum absolute errors (MAE) for both displacement and pressure are given as well. The maximum values for displacement *u* and pressure *p* are $u_{\text {max}}=0.07969$ and $p_{\text {max}}=0.048889$ respectively. The analytical reference solution was computed as explained in the “Methods” section.

First, we performed a logarithmic grid search for the consolidation problem with the fixed values $\alpha =1$, $\beta =1$, as well as a sine loading of $f(t)=\sin \left( \frac{\pi t}{2}\right)$, where $a=1$. The column length was set to $L=1$ and the simulation time is scaled to accommodate the load of the first quarter period of a full load cycle of the sine function, i.e., $t_{\text {end}}=1$. We tested all permutations for the transformation parameters $w_u$, $w_p$, $b_u$, and $b_p$ of the set $\{1\textrm{e}{-}{5}, 1\textrm{e}{-}{4}, 1\textrm{e}{-}{3}, 1\textrm{e}{-}{2}, 1\textrm{e}{-}{1}, 1\textrm{e}{-}{0}, 1\textrm{e}{-}{1}, 1\textrm{e}{-}{2}\}$ resulting in 4096 combinations, where the AfPINN for every combination was trained three times to gather statistics. Thus, a grand total of 12288 training runs were performed. Table 1 shows the best 15 results regarding the ${\mathscr {L}}_2$ relative error of both field variables *u* and *p* after training a single AfPINN with each of these combinations. The ${\mathscr {L}}_2$ relative error is thereby evaluated discretely on the grid of $101\times 101$ points as ${\mathscr {L}}_2({\textbf{x}},\hat{{\textbf{x}}}) = \frac{\vert \vert {\textbf{x}}-\hat{{\textbf{x}}}\vert \vert _2}{\vert \vert {\textbf{x}}\vert \vert _2}$. The worst instances show an ${\mathscr {L}}_2$ relative error of up to 3760.52, showcasing the extremes of the given set with $w_u=1\textrm{e}{-}{5}$, $w_p=1\textrm{e}{-}{5}$, $b_u=1\textrm{e}{-}{2}$, and $b_p=1\textrm{e}{-}{2}$. The relevant deviation always concerns the pressure, which is approximately one order of magnitude higher than the absolute displacement error’s maximum. This corroborates the specific nature of the given problem, where the computation of the pressure is more challenging within the training process, as no value other than 0 is given in all boundary conditions.

**Figure 3:**
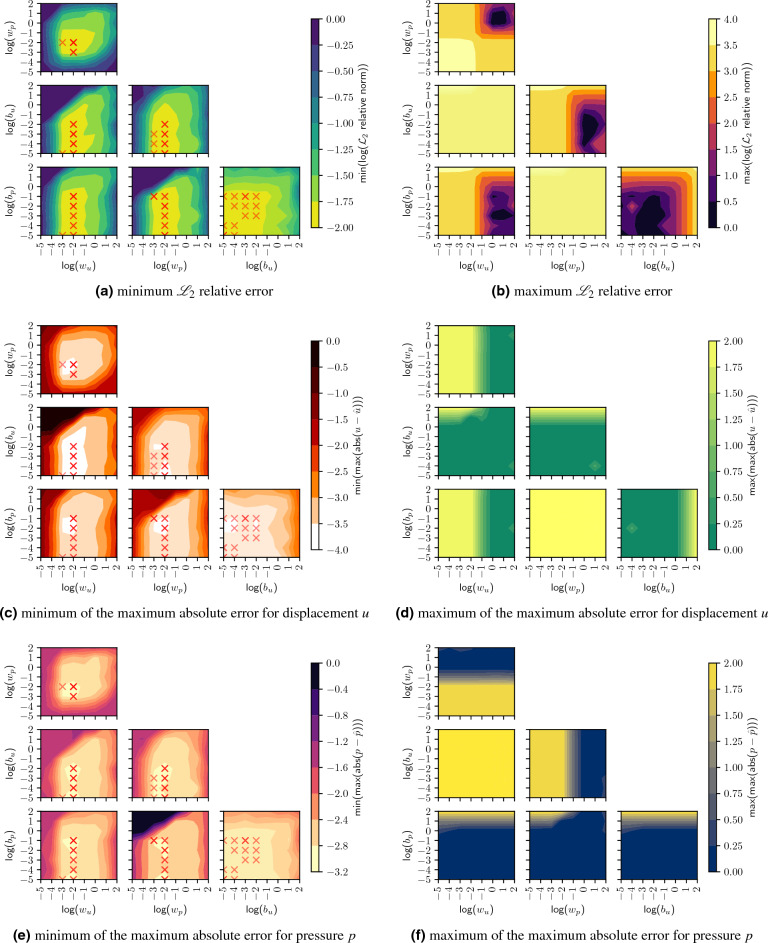
Pairwise contour plots of the minimum and maximum of the ${\mathscr {L}}_2$ relative error as well as the maximum absolute error for displacement *u* and pressure *p* over the tested parameter combinations. The logarithm is used for all quantities for better visualization. Further, the best 15 results as given in Table 1 are marked as red crosses in the plots of minimal values.

We proceed with our analysis of the 5-dimensional data set by looking at the minimum and maximum of the ${\mathscr {L}}_2$ relative error in the pairwise plots of Fig. 3, where the points of the best 15 values according to Table 1 are additionally marked in the contour plots of the minimal error. Figure 3b shows that no choice of only two parameters can force convergence, so a higher-order solution (at least 3 parameters) must be considered. Even the better ranges show relative errors between 32 and 100%. However, all combinations of $b_u$ and $w_u$, as well as $b_p$ and $w_p$ have the potential for complete divergence, while at least high values of the scaling factors $w_u$ and $w_p$ with low values of the respective complementary offset parameter, i.e., $b_p$ and $b_u$, limit the error to the mentioned interval. Similarly, high scaling factors can offset each other, while low offset parameters prevent complete divergence. In contrast, a considerably simpler interpretation is evident for Fig. 3a. In the plots for scaling and offset parameters of the same field variable, there is a range in which even the best value does not converge to a reasonable solution so that high scaling parameters can only compensate high offset parameters. This can be understood as a high offset shifting the values out of the corresponding target range, which only high scaling factors can compensate for. Furthermore, as shown in Fig. 3a and in Table 1, the 15 best values are more restricted in the choice of scaling factors, where $w_u$ and $w_p$ are predominantly set to take the value $1\textrm{e}{-}2$, while $1\textrm{e}{-}3$ has one occurrence each in the best 15 values. The offset factors scatter here to $1\textrm{e}{-}2$, $1\textrm{e}{-}3$, $1\textrm{e}{-}4$, $1\textrm{e}{-}5$ for $b_u$, and additionally to $1\textrm{e}{-}1$ for $b_p$. Moreover, all offset factors below a certain limit (cf., among others, the plot of $b_u$ versus $b_p$  3a) allow for convergence to a good ${\mathscr {L}}_2$ relative error. In contrast, the scaling factors show a lower and upper limit for the stability range (cf., in particular, the plot of $w_u$ versus $w_p$ in Fig. 3a).

These conclusions are supported by studying the maximum absolute errors of displacement (cf. Fig. 3c,d) and pressure (cf. Fig. 3e,f) in a similar way. On the side of the minima, the same trends as for the minimum ${\mathscr {L}}_2$ relative error, especially regarding the position of steep gradients, are discernible. This shared global behavior of the minima shows that the coupled problem is only jointly solvable since, according to the characteristics of the PDE system, only a coupled solution is sufficient. Low absolute errors in pressure coincide with low absolute errors in displacement. This basic characteristic of coupled systems underlines the complexity of solving aggregated loss functions with competing terms by optimization methods since erroneous solutions propagate to other variables and cause the overall system to diverge. Therefore, the analysis cannot be disentangled.

Besides the lower absolute error obtained for the displacement, the favorable range in the pair plot of log($b_u$) and log($w_u$) is significantly wider along log($w_u$) for displacement than for pressure. This shows that the pressure is potentially much more difficult to optimize for, and that in addition to the larger absolute errors, it is also much more difficult to localize the optimal parameter for offset and scaling. This also reflects the basic characteristic of the chosen consolidation problem, where values for the (maximum) displacement are given, while the (maximum) pressure value has to be learned completely from the PDE and the corresponding boundary conditions. Another difference is the position of the largest absolute error on the minimum side. For the displacement, it is located in the pair plot for displacement scaling factor $w_u$ and displacement offset parameter $b_u$, while for the pressure, it is exactly the opposite: The largest value is located in the pair plot for pressure scaling factor $w_p$ and pressure offset parameter $b_p$. Looking at the side of the maxima, the interaction also emerges: Subplot per subplot, the maximum ${\mathscr {L}}_2$ relative error (Fig. 3b) can be composed qualitatively of a superposition of the respective plots of the maximum absolute error for displacement (Fig. 3d) and pressure (Fig. 3f).

As expected, the maximum error for the displacement is independent of the offset and scaling factor of the pressure and vice versa for the pressure and the offset and scaling factors of the displacement. From Fig. 3d, one can derive a lower bound for $w_u$ between $1\textrm{e}{-}1$ and $1\textrm{e}0$ and an upper bound for $b_u$ between $1\textrm{e}0$ and $1\textrm{e}{-}1$, where higher values of $b_u$ can be counterbalanced with high values of $w_u$. Following the same approach, one can analogously derive from Fig. 3f the lower bound for $w_p$ in the same range as $w_u$ and a higher bound for $b_p$ as $b_u$, again allowing higher values of $b_p$ to be balanced with high $w_p$ values. Notably, the bounds for offset and scaling factors are of the same order of magnitude. Here, it seems that there is at least a rough correlation with the maximum field values, or their range, i.e., $u \in [0, u_{\text {max}}=0.07969]$ and $p \in [0, p_{\text {max}}=0.048889]$. Again, the pressure and displacement are of the same order of magnitude and thus only slightly differ. In the logarithmic plots, hardly any differences are discernible when looking at the values at which the gradients between high and low values commence, although a correlation seems natural. Nevertheless, the respective lower and upper bounds to delimit stability areas are missing here. What remains unclear for now is why the areas of stability in the plots of maximum values behave in exactly the opposite way to the areas of good convergence in the plots of minimum values for the scaling factors. High values of the scaling factors provide a limitation of the divergence, but at the same time smaller values are necessary for reliable convergence. The best values on the side of the maximum of the maximum absolute error already show an error of $10^0=1=100\%$ and therefore, no meaningful convergence happens. Thus, it seems that limiting values of the divergence only prevent a deterioration, but do not enable an improvement.

Finally, we can use the minimum plots (cf. Fig. 3a,c,e) to delimit the areas of acceptable convergence or, respectively, of good results. Ranges can be given for the four parameters in which reasonable results can be expected. These ranges are $\text {log}(w_u)=\text {log}(w_p)\in [{-}3.5, {-}1]$ and $\text {log}(b_u)=\text {log}(b_p)\in [{-}5, 0]$. Note that we could hypothetically extend the lower limit of the offset factors towards ${-}\infty$, but we limit ourselves to the previously studied region. We can now deviate from the grid search considering these values and perform a Bayes optimization in this area to obtain the final optimized values.

**Figure 4:**
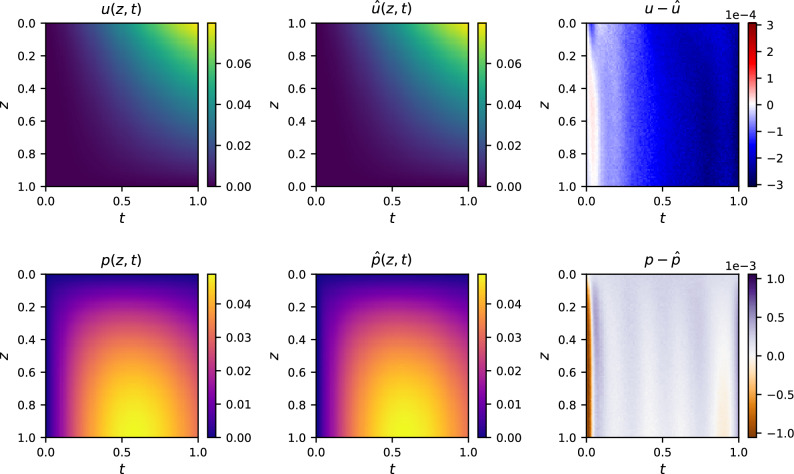
Field plots for a replicate run with the best values from a Bayes optimization in terms of relative ${\mathscr {L}}_2$ error ($w_u=3.18\textrm{e}{-}3$, $w_b=1.26\textrm{e}{-}2$, $b_u=9.86\textrm{e}{-}5$, and $b_p=3.82\textrm{e}{-}4$). Analytically calculated pressure and displacement curves, the AfPINN solution, and the absolute difference between the exact (displacement *u* and pressure *p*) and AfPINN (displacement $\hat{u}$ and pressure $\hat{p}$) solutions are plotted. The values were evaluated on a grid of $101 \times 101$ points resulting in an ${\mathscr {L}}_2$ relative error of $7.50\textrm{e}{-}3$.

We performed a Bayes optimization for the four parameters in the given ranges with scikit-optimize^77^ and its standard settings for Bayes optimization using Gaussian processes with 1000 calls. Updating Table 1 with the new values obtained from Bayes optimization for the scaling, offset parameters, and the ${\mathscr {L}}_2$ relative error as the metric yields Table 2. Even though more values with good ${\mathscr {L}}_2$ relative error are now among the best 30, the final value range is already known from the grid search. A residual variance remains due to the stochastic training properties of ANNs and PINNs. In particular for the scaling factors, the corresponding results are concentrated in or around the interval $[1\textrm{e}{-}3, 1\textrm{e}{-}2]$. Likewise, a stronger scattering of the offset factors is recognizable here, although the interval was also already estimated. Finally, only one run from the Bayes optimization performs better than the previous best value and even falls below an ${\mathscr {L}}_2$ relative error of $1\textrm{e}{-}2$. The corresponding results in form of field plots for displacement *u*, pressure *p*, and the resulting absolute error are shown in Fig. 4. Nevertheless, this improvement can also be attributed to a stochastic effect since no result was obtained from the 999 other values from the Bayesian optimization that also fall below the lowest value from the grid search. To quantify the stochastic effect, 51 additional runs were made with the best values, which yielded a mean ${\mathscr {L}}_2$ relative error of $7.51\textrm{e}{-}3$ with a standard deviation of $3.29\textrm{e}{-}3$. This is a further improvement over the values in Table 2. The plot shown in Fig. 4 was the run closest to the mean yielding a ${\mathscr {L}}_2$ relative error of $7.50\textrm{e}{-}3$.

While the prediction and the analytic solution agree visually, the difference plots show deviations in the expected magnitude (cf. Table 1). Especially in the initial and the closely following time steps, an almost ideal solution is generated for the displacement, while the AfPINN solution predicts somewhat larger values. In addition to the global trend that error increases over time, there is also a trend that errors are propagated from top to bottom according to the force introduced into the system. A different behavior is evident for the pressure field. The AfPINN solution gives a stronger upward-deviating value compared to the analytical solution, especially in the beginning. Based on this, periodic deviations occur where the AfPINN solution is slightly smaller than the analytical solution.

The pressure error is an order of magnitude smaller than the displacement error (see Fig. 4). Nevertheless, the areas of larger errors coincide in the two plots. The area in which the displacement has the greatest accuracy at the first time steps coincides with the area in which the pressure has its greatest deviation. The error increases for larger times. Periodic patterns in one error plot can also be found in the other error plot. Similar patterns were observed in other runs with the same settings.

**Table 2:** The 30 best combinations of scaling ($w_u$ and $w_p$) and offset parameters ($b_u$ and $b_p$) for $\alpha =\beta =1$ based on the $\mathscr {L}_2$ relative error by updating Table 1 with the results from a Bayes optimization.

$w_u$	$w_p$	$b_u$	$b_p$	$\mathscr {L}_2$ rel. err.
$3.18\textrm{e}{-}3$	$1.26\textrm{e}{-}2$	$9.86\textrm{e}{-}5$	$3.82\textrm{e}{-}4$	$8.69\textrm{e}{-}3$
$\underline{1\textrm{e}{-}2}$	$\underline{1\textrm{e}{-}2}$	$\underline{1\textrm{e}{-}5}$	$\underline{1\textrm{e}{-}4}$	$\underline{1.00\textrm{e}{-}2}$
$1.01\textrm{e}{-}3$	$1.03\textrm{e}{-}2$	$2.98\textrm{e}{-}2$	$2.89\textrm{e}{-}3$	$1.04\textrm{e}{-}2$
$2.84\textrm{e}{-}3$	$2\textrm{e}{-}2$	$9.71\textrm{e}{-}4$	$3.22\textrm{e}{-}5$	$1.04\textrm{e}{-}2$
$2.92\textrm{e}{-}2$	$8.12\textrm{e}{-}3$	$2.78\textrm{e}{-}3$	$1.06\textrm{e}{-}1$	$1.04\textrm{e}{-}2$
$1.14\textrm{e}{-}3$	$1.55\textrm{e}{-}2$	$3.22\textrm{e}{-}2$	$3.65\textrm{e}{-}5$	$1.08\textrm{e}{-}2$
$\underline{1\textrm{e}{-}2}$	$\underline{1\textrm{e}{-}2}$	$\underline{1\textrm{e}{-}3}$	$\underline{1\textrm{e}{-}1}$	$\underline{1.11\textrm{e}{-}2}$
$\underline{1\textrm{e}{-}3}$	$\underline{1\textrm{e}{-}2}$	$\underline{1\textrm{e}{-}5}$	$\underline{1\textrm{e}{-}5}$	$\underline{1.14\textrm{e}{-}2}$
$1.11\textrm{e}{-}2$	$1.47\textrm{e}{-}2$	$1.45\textrm{e}{-}4$	$7.55\textrm{e}{-}2$	$1.14\textrm{e}{-}2$
$8.97\textrm{e}{-}3$	$4.58\textrm{e}{-}2$	$2.26\textrm{e}{-}2$	$5.37\textrm{e}{-}5$	$1.15\textrm{e}{-}2$
$\underline{1\textrm{e}{-}2}$	$\underline{1\textrm{e}{-}3}$	$\underline{1\textrm{e}{-}5}$	$\underline{1\textrm{e}{-}1}$	$\underline{1.15\textrm{e}{-}2}$
$9.89\textrm{e}{-}3$	$9.35\textrm{e}{-}3$	$5.80\textrm{e}{-}3$	$1.16\textrm{e}{-}5$	$1.15\textrm{e}{-}2$
$\underline{1\textrm{e}{-}2}$	$\underline{1\textrm{e}{-}2}$	$\underline{1\textrm{e}{-}4}$	$\underline{1\textrm{e}{-}5}$	$\underline{1.15\textrm{e}{-}2}$
$1.64\textrm{e}{-}3$	$5.61\textrm{e}{-}3$	$6.88\textrm{e}{-}4$	$3.43\textrm{e}{-}2$	$1.16\textrm{e}{-}2$
$6.03\textrm{e}{-}3$	$6.45\textrm{e}{-}3$	$1.88\textrm{e}{-}2$	$1.96\textrm{e}{-}4$	$1.17\textrm{e}{-}2$
$\underline{1\textrm{e}{-}2}$	$\underline{1\textrm{e}{-}2}$	$\underline{1\textrm{e}{-}4}$	$\underline{1\textrm{e}{-}2}$	$\underline{1.18\textrm{e}{-}2}$
$9.42\textrm{e}{-}3$	$1.07\textrm{e}{-}2$	$4.33\textrm{e}{-}3$	$8.26\textrm{e}{-}5$	$1.18\textrm{e}{-}2$
$3.68\textrm{e}{-}3$	$6.51\textrm{e}{-}3$	$1.25\textrm{e}{-}2$	$1.18\textrm{e}{-}3$	$1.19\textrm{e}{-}2$
$6.77\textrm{e}{-}3$	$1.03\textrm{e}{-}2$	$1.44\textrm{e}{-}3$	$4.21\textrm{e}{-}5$	$1.19\textrm{e}{-}2$
$8.39\textrm{e}{-}4$	$3.23\textrm{e}{-}2$	$7.52\textrm{e}{-}3$	$2.96\textrm{e}{-}1$	$1.19\textrm{e}{-}2$
$5.62\textrm{e}{-}3$	$3.62\textrm{e}{-}3$	$1.65\textrm{e}{-}5$	$3.25\textrm{e}{-}5$	$1.19\textrm{e}{-}2$
$3.35\textrm{e}{-}3$	$2.86\textrm{e}{-}2$	$4.19\textrm{e}{-}2$	$2.82\textrm{e}{-}2$	$1.20\textrm{e}{-}2$
$\underline{1\textrm{e}{-}2}$	$\underline{1\textrm{e}{-}2}$	$\underline{1\textrm{e}{-}2}$	$\underline{1\textrm{e}{-}3}$	$\underline{1.20\textrm{e}{-}2}$
$5.65\textrm{e}{-}3$	$1.04\textrm{e}{-}2$	$7.53\textrm{e}{-}4$	$1.39\textrm{e}{-}3$	$1.21\textrm{e}{-}2$
$2.51\textrm{e}{-}3$	$4.83\textrm{e}{-}3$	$6.53\textrm{e}{-}5$	$1.98\textrm{e}{-}5$	$1.22\textrm{e}{-}2$
$\underline{1\textrm{e}{-}2}$	$\underline{1\textrm{e}{-}2}$	$\underline{1\textrm{e}{-}3}$	$\underline{1\textrm{e}{-}2}$	$\underline{1.22\textrm{e}{-}2}$
$\underline{1\textrm{e}{-}2}$	$\underline{1\textrm{e}{-}2}$	$\underline{1\textrm{e}{-}5}$	$\underline{1\textrm{e}{-}5}$	$\underline{1.22\textrm{e}{-}2}$
$\underline{1\textrm{e}{-}2}$	$\underline{1\textrm{e}{-}2}$	$\underline{1\textrm{e}{-}2}$	$\underline{1\textrm{e}{-}1}$	$\underline{1.22\textrm{e}{-}2}$
$4.51\textrm{e}{-}3$	$2.47\textrm{e}{-}3$	$5.90\textrm{e}{-}5$	$1.42\textrm{e}{-}3$	$1.22\textrm{e}{-}2$
$3.07\textrm{e}{-}2$	$2.46\textrm{e}{-}3$	$4.39\textrm{e}{-}2$	$3.86\textrm{e}{-}2$	$1.23\textrm{e}{-}2$

Values from the previous grid search are displayed in underline.

### Comparison of standard PINNs and AfPINNs for Biot’s theory

**Figure 5:**
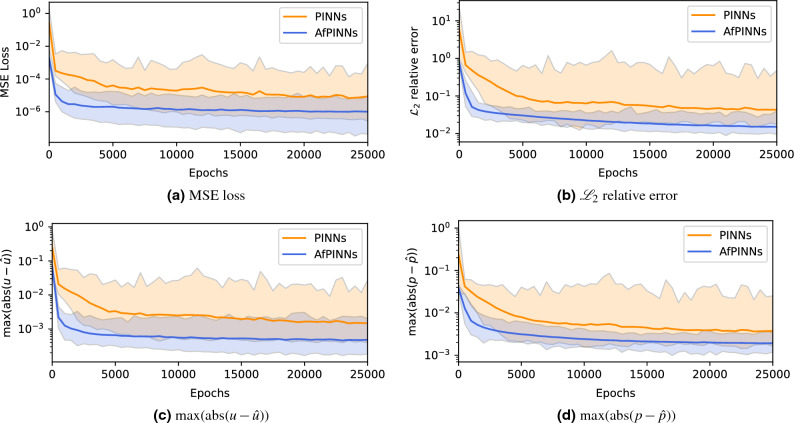
Comparison of AfPINNs and vanilla PINNs for Biot’s theory by mean (thick line), along with minimum and maximum value as shaded area calculated over 500 runs each with intermediate values taken every 500 steps for 25,000 total epochs. MSE Loss, ${\mathscr {L}}_2$ relative error, and maximum of the absolute error for displacement *u* and pressure *p* are plotted with a logarithmic y-scale. It can be seen that AfPINNs perform better on average and have a significantly lower variability than PINNs.

Based on the previous analysis, we can now compare vanilla PINNs, implemented by setting scaling parameter equal to 1 and offset parameter equal to 0, with AfPINNs. As argued in the previous section, $w_u=1\textrm{e}{-}2$ and $w_p=1\textrm{e}{-}2$ are reasonable assumptions since the influence of the offset factors is smaller as long as they are below a certain limit. There is no detriment from choosing them to be in the same order of magnitude as the scaling values, so we have decided on $b_u=1\textrm{e}{-}2$ and $b_p=1\textrm{e}{-}2$ here for further analysis. For both variants, 500 instances were trained while saving loss, ${\mathscr {L}}_2$ relative error, and the maximum absolute error of u and p every 500 epochs. Based on this, the average value in each epoch, as well as the minimum and maximum value for PINNs and AfPINNs, can now be specified for each variable. Figure 5 contains the corresponding plots, where for each time mean, minimum, and maximum value of the 500 runs are shown.

Considering all four quantities (MSE loss in Fig. 5a, ${\mathscr {L}}_2$ relative error in Fig. 5b, MAE of displacement *u* in Fig. 5c, and MAE of pressure *p* in Fig. 5d), AfPINNs do not only achieve lower, i.e., better mean value at all stages compared to PINNs but the distance between the curves of AfPINNs and vanilla PINNs is constant on a logarithmic scale. Further, the mean of AfPINNs after 25,000 epochs is in a similar range as the lowest value of a vanilla PINN, while AfPINNs have a smaller range between lowest and highest values at all stages, which is particularly evident for the ${\mathscr {L}}_2$ relative error in Fig. 5b. Furthermore, the mean value of the pressure MAE is higher than the mean value of the displacement MAE for both PINNs and AfPINNs. The same applies for the upper and lower bounds for each field quantity. If vanilla PINNs reach a certain value, AfPINNs have reached this value earlier on average, i.e., with a lower number of training epochs. Due to the discrete steps of 500 epochs, a strong kink appears after the initial 500 steps, after which improvement gradually flattens. In addition, we compared the 1000 collocation points we chose as default to PINNs and AfPINNs with 100 and 10,000 collocation points. The plots in Fig. S.4 in the supplementary material show that the number of collocation points has no discernible influence on statistical average over 500 runs.

The results we obtained for Biot’s theory and later also the TPM are in good agreement with the results we obtained for the Burgers’ equation, which are presented in the supplementary material in section A. First, also in the plots for MSE Loss, ${\mathscr {L}}_2$ relative error, and maximum of the absolute error of the field variable we can observe the identical tendency that AfPINNs reach significantly smaller values (cf. Fig. S.2). Both the mean value is smaller and the scatter in the min-max range is same or smaller, even though this is somewhat concealed by the logarithmic y-axis. Further, Fig. S.3 shows an example result for Burgers’ equation with an AfPINN.

Based on these impressions, we can now consider precise numerical values. For the four values (MSE loss, maximum absolute deviation in pressure and displacement, and ${\mathscr {L}}_2$ relative error), we consider the mean (Table S.1a) and standard deviation (Table S.1b) of the 500 runs each, the resulting improvement of AfPINNs over PINNs (Table 3), and how many epochs it takes to fall below the given values (Table 4). Looking first at the MSE loss, for normal PINNs the standard deviation is an order of magnitude larger than the corresponding mean value. Consequently, the mean value for the AfPINNs is an order of magnitude lower overall and the standard deviation is in the same order of magnitude as the mean value. In addition, even before a training epoch has finished, AfPINNs show a significantly smaller MSE loss than vanilla PINNs. Thus, AfPINNs start at a point with a lower loss with respect to optimization and exhibit significantly lower stochastic fluctuations, as shown by the standard deviation, during training. For the maximum absolute deviation of both fields, both AfPINNs and normal PINNs exhibit a standard deviation in the dimension of the mean value. Hence, AfPINNs produce better initial value on average as well as lower values in general, the distance between AfPINNs and PINNs for the displacement being just under half an order of magnitude, while this is approximately halved for the pressure within the same order of magnitude. The standard deviation of the pressure is slightly larger than the standard deviation of the displacement for AfPINNs, as shown in Table S.1b. After 25,000 epochs, this results in a mean ${\mathscr {L}}_2$ relative error of $1.51\textrm{e}{-}2$ for AfPINNs versus the $4.30\textrm{e}{-}2$ value for classical PINNs. We can now calculate the percentage improvements for each epoch based on Tables S.1a and S.1b as $\frac{x_{\text{old}}-x_{\text{new}}}{x_{\text{old}}}$. Statements about the improvement of the mean and the improvements of the standard deviations can be given. These improvements, given in percentages, are shown in Table 3. For all four metrics, the improvements in both mean and standard deviation, the values decrease with an increasing number of epochs, but significant improvements remain. After 25,000 epochs, there are mean improvements of $88.41\%$ for the MSE loss, $67.39\%$ for the maximum absolute error of displacement *u*, $48.50\%$ for the maximum absolute error of pressure *p*, and $64.84\%$ for the ${\mathscr {L}}_2$ relative error for AfPINNs over vanilla PINNs.

The number of epochs necessary on average to fall below a given fixed value are shown in Table 4. Since the metrics were evaluated every 500 epochs during the training, only multiples of 500 are displayed in the table. Two effects are visible here despite the residual fuzziness due to the discrete log step size. AfPINNs reach value thresholds at a significantly lower number of epochs than PINNs, and AfPINNs reach values which PINNs cannot. Not only the initial values of AfPINNs are offset, but also vanilla PINNs need significantly longer for individual increments. For example, the transition in the maximum absolute error of the pressure between $7.5\textrm{e}{-}3$ and $5\textrm{e}{-}3$ can be used for a comparison. The AfPINNs need on average approximately 1000 iterations to accomplish this transition, while vanilla PINNs need approximately 8500 epochs. Further, AfPINNs need another 7500 epochs to reach $2.5\textrm{e}{-}3$, while vanilla PINNs do not, although there are still 12,000 iterations left in the training process. Following the reasoning in the explanation for affine transformations, we kept the learning rate fixed in all quantities as not to mix effects of learning rate changes and changes in transformation parameters. Other particularly strong examples of such divergences, where significantly more steps and thus significantly more computational resources must be expended, are found in the MSE loss at the transition from $2.5\textrm{e}{-}5$ to $1.0\textrm{e}{-}5$, and in the ${\mathscr {L}}_2$ relative error at the changes from $2.5\textrm{e}{-}1$ to $1.0\textrm{e}{-}1$, as well as $7.5\textrm{e}{-}2$ to $5.0\textrm{e}{-}2$. Likewise, it is evident in the maximum absolute displacement that AfPINNs produce significantly lower errors and reach errors comparable to normal PINNs after 500 epochs already. Given these results, we conclude that affine transformations accelerate the training of PINNs.

**Table 3:** Improvement of mean and standard deviation of 500 AfPINN runs over 500 PINN runs for MSE Loss, ${\mathscr {L}}_2$ relative error, and maximum of the absolute error for displacement *u* and pressure *p* every 5000 epochs as percentages for Biot’s theory.

Epochs	MSE loss		$\text {max}(|u-\hat{u}|)$		$\text {max}(|p-\hat{p}|)$		${\mathscr {L}}_2$ relative error	
	Mean (%)	SD (%)	Mean (%)	SD (%)	Mean (%)	SD (%)	Mean (%)	SD (%)
0	$99.30$	$99.94$	$72.36$	$98.46$	$83.79$	$98.60$	$86.96$	$99.01$
5000	$94.97$	$97.75$	$80.37$	$90.14$	$60.77$	$83.66$	$68.38$	$93.37$
10,000	$93.12$	$96.90$	$77.59$	$90.65$	$53.15$	$91.36$	$64.59$	$95.25$
15,000	$91.61$	$97.63$	$72.96$	$88.25$	$52.66$	$88.92$	$65.85$	$93.34$
20,000	$86.95$	$94.29$	$69.93$	$84.05$	$48.70$	$87.60$	$65.08$	$92.37$
25,000	$88.41$	$97.59$	$67.39$	$84.56$	$48.50$	$85.03$	$64.84$	$89.75$

**Table 4:** Number of epochs necessary for falling below given values for the mean of 500 PINN and 500 AfPINN runs of the MSE Loss, ${\mathscr {L}}_2$ relative error, and maximum of the absolute error for displacement *u* and pressure *p* for Biot’s theory.

Value	MSE Loss		$\text {max}(|u-\hat{u}|)$		$\text {max}(|p-\hat{p}|)$		${\mathscr {L}}_2$ relative error	
	PINN	AfPINN	PINN	AfPINN	PINN	AfPINN	PINN	AfPINN
$5.0\textrm{e}{-}1$	0	0	0	0	0	0	1000	500
$2.5\textrm{e}{-}1$	500	0	500	0	0	0	2500	500
$1.0\textrm{e}{-}1$	500	0	500	0	500	0	4500	1000
$7.5\textrm{e}{-}2$	500	0	500	0	500	0	6500	1000
$5.0\textrm{e}{-}2$	500	0	500	500	500	0	17,500	1500
$2.5\textrm{e}{-}2$	500	0	500	500	1500	500	–	8000
$1.0\textrm{e}{-}2$	500	0	2500	500	4000	1000	–	–
$7.5\textrm{e}{-}3$	500	0	3000	500	5500	1000	–	–
$5.0\textrm{e}{-}3$	500	0	3500	500	13,000	2000	–	–
$2.5\textrm{e}{-}3$	500	0	10,000	500	–	9500	–	–
$1.0\textrm{e}{-}3$	500	500	–	2000	–	–	–	–
$7.5\textrm{e}{-}4$	500	500	–	3000	–	–	–	–
$5.0\textrm{e}{-}4$	500	500	–	16,500	–	–	–	$\text {-}$
$2.5\textrm{e}{-}4$	1000	500	–	–	–	–	–	–
$1.0\textrm{e}{-}4$	3000	500	–	–	–	–	–	–
$7.5\textrm{e}{-}5$	4000	500	–	–	–	–	–	–
$5.0\textrm{e}{-}5$	4000	500	–	–	–	–	–	–
$2.5\textrm{e}{-}5$	6500	500	–	–	–	–	–	–
$1.0\textrm{e}{-}5$	19,000	1000	–	–	–	–	–	–
$7.5\textrm{e}{-}6$	23,500	1000	–	–	–	–	–	–
$5.0\textrm{e}{-}6$	–	1000	–	–	–	–	–	–
$2.5\textrm{e}{-}6$	–	2500	–	–	–	–	–	–
$1.0\textrm{e}{-}6$	–	–	–	–	–	–	–	–

The values in the training of the networks are recorded every 500 epochs. Accordingly, the mean values are also taken only every 500 epochs, so that only multiples of 500 occur.

### Comparison of standard PINNs and AfPINNs for the theory of porous media

**Figure 6:**
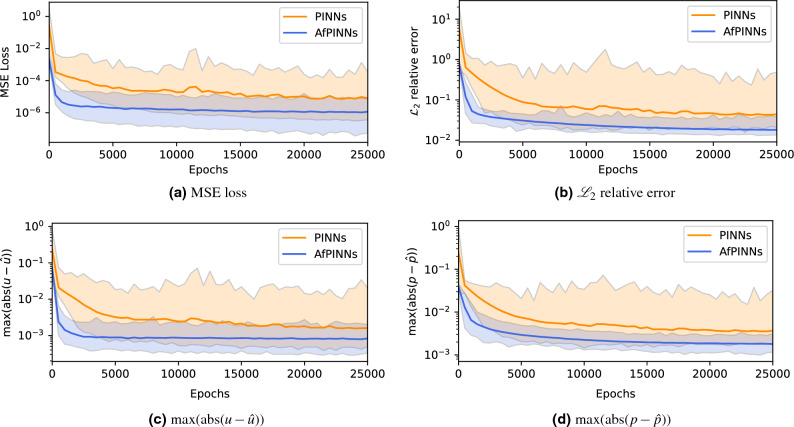
Comparison of AfPINNs and vanilla PINNs for TPM by mean (thick line), as well as minimum and maximum value as shaded area calculated over 500 runs each with intermediate values taken every 500 steps for 25,000 total epochs. MSE Loss, ${\mathscr {L}}_2$ relative error, and maximum of the absolute error for displacement *u* and pressure *p* are plotted with logarithmic *y*-axis.

Starting from the analysis of Biot’s theory, we take identical affine transformation parameters ($w_u=w_p=b_u=b_p=1\textrm{e}{-}2$) and transfer them directly to AfPINNs for solving the TPM formulation of the consolidation problem for the same material and boundary values described in the “Methods” section. We base our considerations here on the closeness and fundamentally similar structure of the equations with additional, albeit numerically weaker terms in the TPM (see Fig. S.1 in the supplementary material, which shows a comparison between the results from both theories for the given values). Again, we compare 500 training runs between vanilla PINNs and AfPINNs with loss, ${\mathscr {L}}_2$ relative error, and maximum absolute errors in displacement and pressure, shown in Fig. 6. The loss and error plots for TPM (Fig. 6) and Biot’s theory (Fig. 5) show nearly identical global behavior. Again, the curves of the AfPINNs are oriented at the lower bounds of the values obtainable by vanilla PINNs. Likewise, the loss and the maximum absolute error of the displacement flattens out very quickly, while the optimization focus changes to the maximum absolute error of the displacement and thus also the relative ${\mathscr {L}}_2$ error. In this case, the logarithmic distance between AfPINNs and vanilla PINNs is almost constant. Due to the similarity of the equations and identical transformation parameters, the global behavior and the values reached are likely to be similar. Likewise, we have not been able to identify any noteworthy influence of the number of collocation points for the TPM (cf. Fig. S.5 in the supplementary material) although we compare across orders of magnitude in terms of numbers, which we have already not been able to establish for Biot’s theory  (cf. Fig. S.4 in the supplementary material).

The plot of the maximum absolute error of the displacement (Fig. 6c) seems to show a constant lower bound for the mean as $1\textrm{e}{-}3$, which is reached relatively early. Accordingly, the evaluation is analogous to that for Biot’s theory, so we study particular numerical values based on improving values and falling below given thresholds regarding the number of epochs to achieve these. Table S.2a shows the mean, Table S.2b shows the standard deviation (both in the supplementary material), and Table 5 shows the resulting improvements for the TPM formulation. We refrain from evaluating a table akin to Table 4 since there are no relevant changes compared to Biot’s theory. Given Table 5, we can quantify an average improvement of roughly $50\%$ in pressure and displacement after 25,000 epochs. In combination, this leads to a mean relative ${\mathscr {L}}_2$ error improvement of $58.80\%$ after 25,000 epochs while decreasing the standard deviation by $90.53\%$. The largest improvement is achieved for the loss. However, the training loss itself is never a suitable metric for determining the quality of a model, since it was specifically used as the minimization goal for the training. Hence, other metrics should be employed to quantify the approximation quality. Here, we decided to use the ${\mathscr {L}}_2$ relative error and the maximal differences between ground truth field values and their approximations.

**Table 5:** Improvement of mean and standard deviation of 500 AfPINN runs over 500 PINN runs for MSE Loss, ${\mathscr {L}}_2$ relative error, and maximum of the absolute error for displacement *u* and pressure *p* every 5000 epochs as percentages for TPM.

Epochs	MSE loss		$\text {max}(|u-\hat{u}|)$		$\text {max}(|p-\hat{p}|)$		${\mathscr {L}}_2$ relative error	
	Mean (%)	SD (%)	Mean (%)	SD (%)	Mean (%)	SD (%)	Mean (%)	SD (%)
0	$99.18$	$99.94$	$68.95$	$98.62$	$84.10$	$98.58$	$86.65$	$99.00$
5000	$94.01$	$96.16$	$72.30$	$87.97$	$60.60$	$83.99$	$65.06$	$92.39$
10,000	$91.46$	$96.09$	$64.37$	$86.09$	$54.41$	$88.45$	$59.82$	$92.32$
15,000	$92.07$	$97.09$	$60.45$	$88.38$	$55.46$	$91.28$	$62.97$	$94.74$
20,000	$89.36$	$94.59$	$55.50$	$81.68$	$51.09$	$84.33$	$60.07$	$90.83$
25,000	$85.60$	$94.68$	$48.26$	$80.87$	$50.97$	$87.18$	$58.80$	$90.53$

## Conclusions

AfPINNs consistently outperform standard PINNs on the coupled problems presented here both in training time and approximation quality. The scaling and offset factors have to be found by a feasible search strategy, e.g., a combination of grid and Bayesian search. Unfortunately, no direct correlations between the factors and the order of magnitudes of the problem parameters were found. For Biot’s theory, the average improvement reaches $64.84\%$, while a transfer approach of the TPM using the same parameters and factors showed an improvement of up to $58.80\%$ in the ${\mathscr {L}}_2$ relative error after 25,000 epochs. Furthermore, the standard deviation is improved by $89.75\%$ for Biot’s theory and by $90.53\%$ for TPM given the transfer approach.

We have provided empirical evidence for the feasibility and usefulness of using affine transformations in PINNs and presented some reasoning as to why this makes sense. The approach helps to alleviate issues arising in unbalanced optimization problems with competing terms in the aggregated loss functions and internal covariate shifts. Many details remain to be fully elucidated, such as whether a faster approximation scheme for the scaling and offset factors using the problem parameters can be found. The underlying theoretical work needs improvement and a proper foundation. Analytical or classical numerical solutions are currently preferable for relatively simple toy problems. Larger, more complex problems may profit from the improved properties of AfPINNs. We did not yet consider second-order optimization methods, such as limited-memory Broyden–Fletcher–Goldfarb–Shanno as sometimes used in PINNs (cf. Karniadakis et al.^37^). Nevertheless, this work showed that transferring the concept of PINNs to strongly coupled problems is possible.

In conclusion, we showed that AfPINNs can significantly reduce the training time and that the choice of parameters also improve the general convergence behavior significantly, without resorting to complex training or sampling schemes.

### Supplementary Information

Supplementary Information.

## Data Availability

Data underlying the results presented in this paper are not publicly available at this time but may be obtained from the corresponding author upon reasonable request.
