# Supplementary material for: Affine transformations accelerate the training of physics-informed neural networks of a one-dimensional consolidation problem
Source: Sci Rep. 2023 Sep 20;13:15566. doi: 10.1038/s41598-023-42141-x (PMC10511457; doi:10.1038/s41598-023-42141-x)
Supplement: Supplementary file 1 — Supplementary Information. [file 41598_2023_42141_MOESM1_ESM.pdf]

Affine Transformations Accelerate the Training of Physics-Informed Neural Networks of a One-Dimensional Consolidation Problem

Luis Mandl[1], André Mielke[1], Seyed Morteza Seyedpour[1,2], and Tim Ricken[1,2,*]

[1]Institute of Structural Mechanics and Dynamics in Aerospace Engineering, Faculty of Aerospace Engineering and Geodesy, University of Stuttgart, Pfaffenwaldring 27, 70569 Stuttgart, Germany
[2]Biomechanics Lab, Institute of Structural Mechanics and Dynamics in Aerospace Engineering, Faculty of Aerospace Engineering and Geodesy, University of Stuttgart, Pfaffenwaldring 27, 70569 Stuttgart, Germany
[*]tim.ricken@isd.uni-stuttgart.de

Supplementary Material

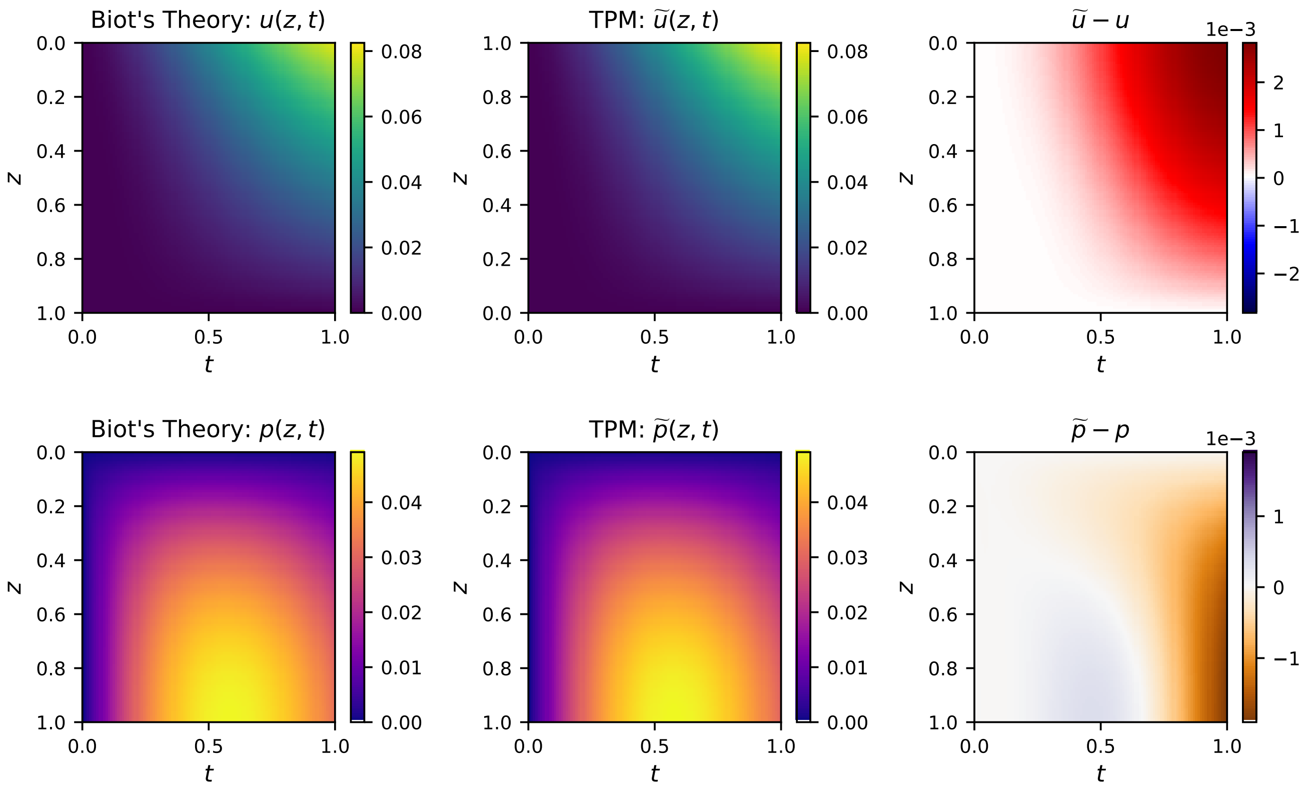

Figure S.1. Comparison of the one-dimensional consolidation problem using Biot's theory and TPM with $\alpha = 1$, $\beta = 1$, and $a = 0.1$ on $(z,t) \in [0\text{ m},\ 1\text{ m}] \times [0\text{ s},\ 1\text{ s}]$ and 101×101 sampling points.

	MSE Loss		$\max(\lvert u-\hat{u}\rvert)$		$\max(\lvert p-\hat{p}\rvert)$		$\mathscr{L}_2$ relative error	
Epochs	PINN	AfPINN	PINN	AfPINN	PINN	AfPINN	PINN	AfPINN
0	2.60e - 1	1.83e - 3	2.52e - 1	6.97e - 2	2.41e - 1	3.90e - 2	5.76	7.52e - 1
5000	4.04e - 5	2.03e - 6	3.36e - 3	6.59e - 4	7.95e - 3	3.12e - 3	9.58e - 2	3.03e - 2
10000	1.94e - 5	1.33e - 6	2.49e - 3	5.57e - 4	5.15e - 3	2.41e - 3	6.32e - 2	2.24e - 2
15000	1.56e - 5	1.31e - 6	1.96e - 3	5.29e - 4	4.47e - 3	2.12e - 3	5.44e - 2	1.86e - 2
20000	8.21e - 6	1.07e - 6	1.65e - 3	4.96e - 4	3.89e - 3	2.00e - 3	4.65e - 2	1.62e - 2
25000	8.73e - 6	1.01e - 6	1.48e - 3	4.82e - 4	3.73e - 3	1.92e - 3	4.30e - 2	1.51e - 2

(a) mean

	MSE Loss		$\max(\lvert u-\hat{u}\rvert)$		$\max(\lvert p-\hat{p}\rvert)$		$\mathscr{L}_2$ relative error	
Epochs	PINN	AfPINN	PINN	AfPINN	PINN	AfPINN	PINN	AfPINN
0	3.15e - 1	1.91e - 4	1.45e - 1	2.22e - 3	1.47e - 1	2.06e - 3	3.11	3.09e - 2
5000	1.01e - 4	2.26e - 6	2.96e - 3	2.92e - 4	3.35e - 3	5.48e - 4	6.64e - 2	4.40e - 3
10000	4.12e - 5	1.28e - 6	2.64e - 3	2.47e - 4	4.21e - 3	3.64e - 4	6.83e - 2	3.24e - 3
15000	5.31e - 5	1.26e - 6	2.19e - 3	2.57e - 4	3.02e - 3	3.35e - 4	5.18e - 2	3.45e - 3
20000	1.96e - 5	1.12e - 6	1.72e - 3	2.75e - 4	2.54e - 3	3.15e - 4	4.15e - 2	3.16e - 3
25000	4.41e - 5	1.07e - 6	1.68e - 3	2.60e - 4	2.01e - 3	3.01e - 4	3.17e - 2	3.25e - 3

(b) standard deviation

Table S.1. Mean and standard deviation of 500 PINN and AfPINN runs for MSE Loss, $\mathscr{L}_2$ relative error, and maximum of the absolute error for displacement u and pressure p every 5000 epochs as absolute values for Biot's Theory.

	MSE Loss		$\max(\lvert u-\hat{u}\rvert)$		$\max(\lvert p-\hat{p}\rvert)$		$\mathscr{L}_2$ relative error	
Epochs	PINN	AfPINN	PINN	AfPINN	PINN	AfPINN	PINN	AfPINN
0	2.40e - 1	1.96e - 3	2.34e - 1	7.26e - 2	2.46e - 1	3.91e - 2	5.64	7.53e - 1
5000	3.32e - 5	1.99e - 6	3.20e - 3	8.88e - 4	7.44e - 3	2.93e - 3	8.80e - 2	3.08e - 2
10000	1.88e - 5	1.61e - 6	2.44e - 3	8.69e - 4	4.92e - 3	2.24e - 3	5.93e - 2	2.38e - 2
15000	1.57e - 5	1.24e - 6	2.14e - 3	8.47e - 4	4.40e - 3	1.96e - 3	5.54e - 2	2.05e - 2
20000	1.04e - 5	1.10e - 6	1.81e - 3	8.04e - 4	3.81e - 3	1.86e - 3	4.66e - 2	1.86e - 2
25000	8.06e - 6	1.16e - 6	1.60e - 3	8.28e - 4	3.67e - 3	1.80e - 3	4.40e - 2	1.81e - 2

(a) mean

	MSE Loss		$\max(\lvert u-\hat{u}\rvert)$		$\max(\lvert p-\hat{p}\rvert)$		$\mathscr{L}_2$ relative error	
Epochs	PINN	AfPINN	PINN	AfPINN	PINN	AfPINN	PINN	AfPINN
0	2.90e - 1	1.83e - 4	1.36e - 1	1.87e - 3	1.44e - 1	2.05e - 3	2.88	2.87e - 2
5000	5.53e - 5	2.12e - 6	2.42e - 3	2.91e - 4	3.02e - 3	4.84e - 4	4.96e - 2	3.77e - 3
10000	4.08e - 5	1.60e - 6	2.10e - 3	2.92e - 4	3.09e - 3	3.57e - 4	4.66e - 2	3.58e - 3
15000	4.27e - 5	1.24e - 6	2.34e - 3	2.72e - 4	3.45e - 3	3.01e - 4	5.57e - 2	2.93e - 3
20000	2.28e - 5	1.23e - 6	1.52e - 3	2.79e - 4	1.86e - 3	2.91e - 4	3.11e - 2	2.86e - 3
25000	2.21e - 5	1.17e - 6	1.56e - 3	2.98e - 4	2.21e - 3	2.83e - 4	3.44e - 2	3.26e - 3

(b) standard deviation

Table S.2. Mean and standard deviation of 500 PINN and AfPINN runs for MSE Loss, $\mathscr{L}_2$ relative error, and maximum of the absolute error for displacement u and pressure p every 5000 epochs as absolute values for TPM.

A AfPINNs for Burgers' Equation

To supplement the consolidation problem with two theories described in the main body of the paper, we also studied the original example with the Burgers' equation from the paper by Raissi et al.[1] which is also part of the examples of DeepXDE[2]. The problem is governed by the Burgers' equation in one spatial dimension with the following Dirichlet BCs

$$\frac{\partial u(x,t)}{\partial t} + u(x,t)\frac{\partial u(x,t)}{\partial x} - \frac{0.01}{\pi}\frac{\partial^2 u(x,t)}{\partial x^2} = 0, \tag{S.1}$$

$$u(0,x) = -\sin(\pi x), \tag{S.2}$$

$$u(t,-1) = u(t,1) = 0, \tag{S.3}$$

with fluid velocity $u(x,t)$ in the spatiotemporal domain given by $x \in [-1\text{ m},\ 1\text{ m}]$ and $t \in [0\text{ s},\ 1\text{ s}]$. We adopted the default values of DeepXDE for our hyperparameters, but increased the number of collocation points by a factor of 10 to more sharply constrain the position of the discontinuity in the solution and thus not randomly increase the $\mathscr{L}_2$ relative error by a detrimental choice of collocation points. Hence, we used 80 sample points on the BC, 160 sample points on the IC, and 25400 collocation points. The network architecture consists of 2 input neurons in the input layer (x and t), 3 hidden layers with 20 neurons and a nonlinear *tanh* activation, as well as 1 output neuron (u) with linear activation. We used ADAM optimization[3] with 15000 epochs and a fixed learning rate of 10^{-3} on the full data batch. All weights and biases were initialized using the Glorot normal initializer[4]. A Bayes optimization with 300 calls yielded the parameters of the affine transformation to $w_u = 0.1182153$ and $b_u = 0.0025055$ with a $\mathscr{L}_2$ relative error of 3.67e - 3. Given those parameters of the affine transformation, we were able to compare vanilla PINNs with identical training and network settings to AfPINNs with the respective affine layer. The results of this comparison are shown in figure S.2. Furthermore, figure S.3 depicts the solution of the given problem with an arbitrary AfPINN given the above parameters of the affine transformation compared to the analytical solution. The results are in good agreement with our findings for the coupled problem of consolidation.

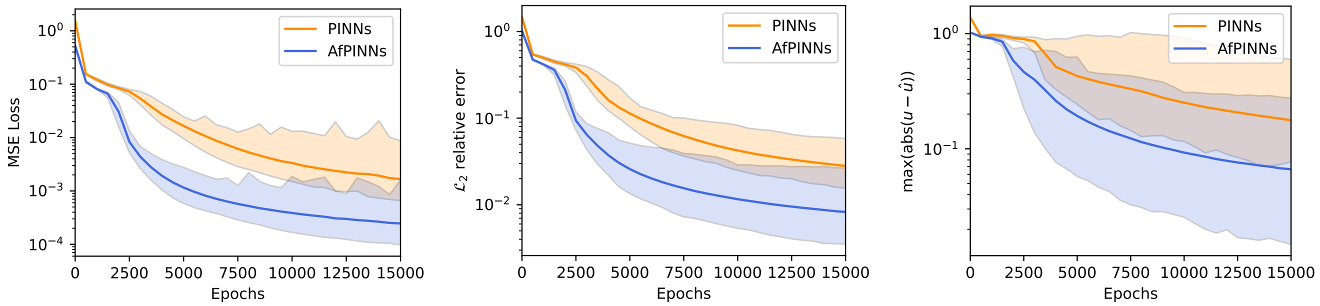

(a) MSE loss **(b)** $\mathscr{L}_2$ relative error PINNs **(c)** max(abs($u - \hat{u}$))

Figure S.2. Comparison of AfPINNs and vanilla PINNs for Burgers' equation by mean (thick line), along with minimum and maximum value as shaded area calculated over 500 runs each with intermediate values taken every 500 steps for 15000 total epochs. MSE Loss, $\mathscr{L}_2$ relative error, and maximum of the absolute error for fluid speed u are plotted with a logarithmic y-scale. It can be seen that AfPINNs perform better on average and have a significantly lower variability than PINNs.

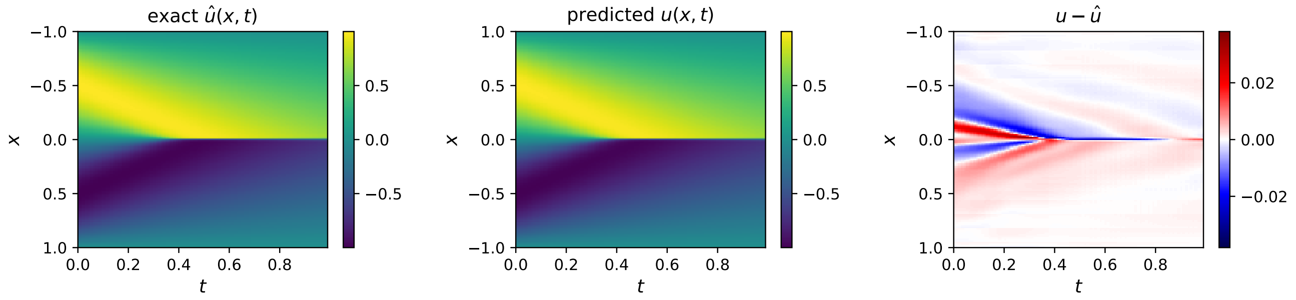

Figure S.3. Result of a single AfPINN compared to the analytical solution for Burgers' equation with a $\mathscr{L}_2$ relative error of 6.92e - 3.

B Influence of Collocation Points for AfPINNs and the One-Dimensional Consolidation Problem

In addition to the results obtained in the main part of this work with 1000 collocation points, we compared the performance of AfPINNs with 100 and 10000 collocation points. For this, all other hyperparameters were left unchanged including the affine weights obtained, i.e., $w_u = w_p = b_u = b_p = 1\mathrm{e}-2$. as well as the 100 sample points on the BCs and 100 sample points on the ICs, respectively. Figure S.4 shows the results for Biot's theory, while figure S.5 depicts the same for TPM. In both cases, it can be shown that the number of collocation points has only a marginal if not completely negligible influence for both vanilla PINNs and AfPINNs.

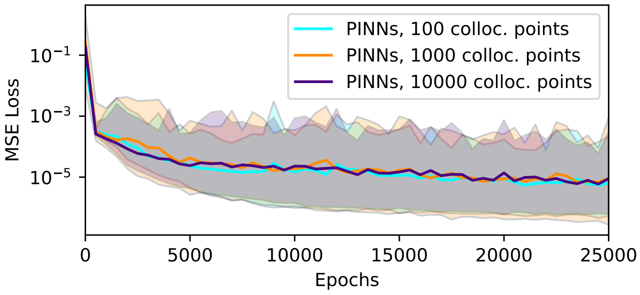

(a) MSE loss PINNs

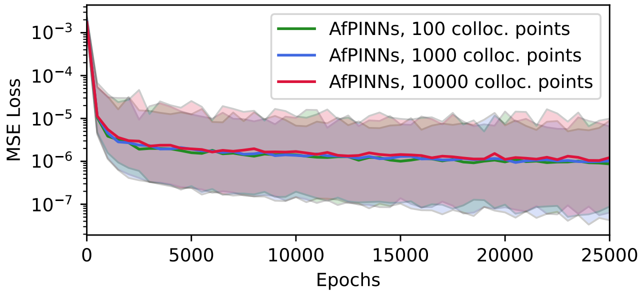

(b) MSE loss AfPINNs

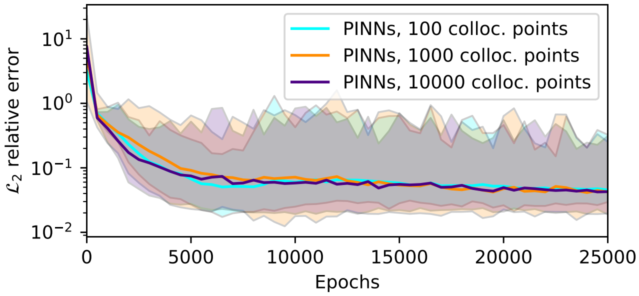

(c) $\mathscr{L}_2$ relative error PINNs

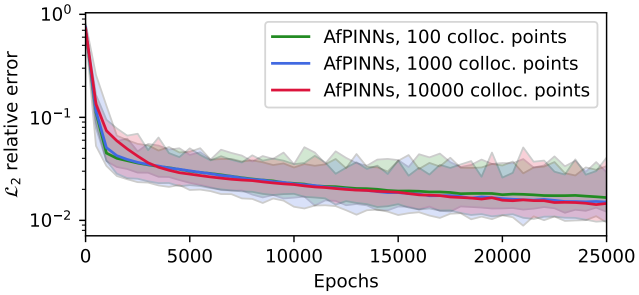

(d) $\mathscr{L}_2$ relative error AfPINNs

Figure S.4. Comparison of AfPINNs and vanilla PINNs for Biot's theory by mean (thick line), along with minimum and maximum value as shaded area calculated over 250 runs each with intermediate values taken every 500 steps for 25000 total epochs. MSE Loss and $\mathscr{L}_2$ relative error are plotted with a logarithmic y-scale for 100, 1000, and 10000 collocation points respectively.

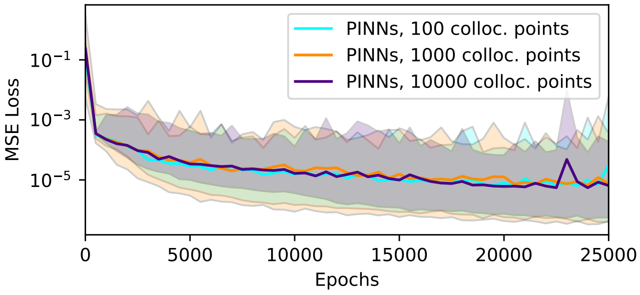

(a) MSE loss PINNs

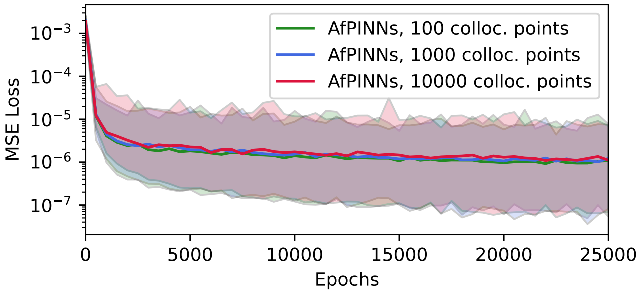

(b) MSE loss AfPINNs

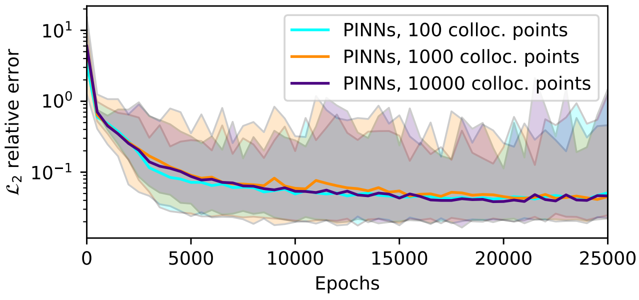

(c) $\mathscr{L}_2$ relative error PINNs

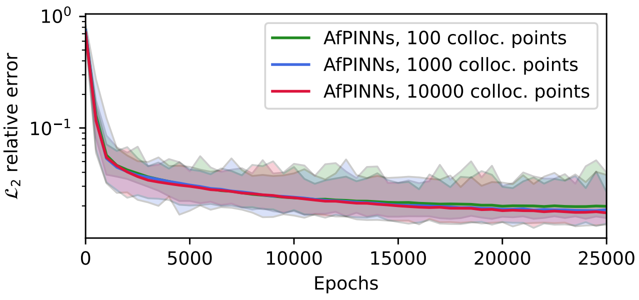

(d) $\mathscr{L}_2$ relative error AfPINNs

Figure S.5. Comparison of AfPINNs and vanilla PINNs for TPM by mean (thick line), along with minimum and maximum value as shaded area calculated over 250 runs each with intermediate values taken every 500 steps for 25000 total epochs. MSE Loss and $\mathscr{L}_2$ relative error are plotted with a logarithmic y-scale for 100, 1000, and 10000 collocation points respectively.

References

1. Raissi, M., Perdikaris, P. & Karniadakis, G. Physics-informed neural networks: A deep learning framework for solving forward and inverse problems involving nonlinear partial differential equations. *J. Comput. Phys.* **378**, 686–707, DOI: 10.1016/j.jcp.2018.10.045 (2019).

2. Lu, L., Meng, X., Mao, Z. & Karniadakis, G. E. Deepxde: A deep learning library for solving differential equations. *SIAM Rev.* **63**, 208–228, DOI: 10.1137/19m1274067 (2021).

3. Kingma, D. P. & Ba, J. Adam: A method for stochastic optimization (2014). arXiv:1412.6980.

4. Glorot, X. & Bengio, Y. Understanding the difficulty of training deep feedforward neural networks. In Teh, Y. W. & Titterington, M. (eds.) *Proceedings of the Thirteenth International Conference on Artificial Intelligence and Statistics*, vol. 9 of *Proceedings of Machine Learning Research*, 249–256 (PMLR, Chia Laguna Resort, Sardinia, Italy, 2010).
